# A Comparative Study to Evaluate the Renoprotective Effect of Antidiabetic Drugs in Patients With Type 2 Diabetes Mellitus

**DOI:** 10.7759/cureus.113475

**Published:** 2026-07-27

**Authors:** Meenu Pichholiya, Raj Abhinav, Sakshi Singh, Arvind K Yadav, Sangita Gupta

**Affiliations:** 1 Pharmacology, American International Institute of Medical Sciences, Udaipur, IND; 2 Pharmacology, Geetanjali Medical College and Hospital, Geetanjali University, Udaipur, IND

**Keywords:** antidiabetic drugs, oral hypoglycemic agents, renal function test, renoprotective, type 2 diabetes mellitus

## Abstract

Introduction: Type 2 diabetes mellitus (T2DM) is a metabolic condition that often leads to long-term microvascular and macrovascular complications. Among its most frequent and severe complications is diabetic nephropathy, which stands as a primary contributor to chronic kidney disease and end-stage renal disease on a global scale. Literature search studies have exhibited both renoprotective and non-renoprotective effects of different antidiabetic drugs, and to our knowledge, no study comparing the renoprotective effect of antidiabetic drugs was found, so this study was designed to assess the impact of these drugs on kidney function in patients with T2DM.

Materials and methods: A prospective observational study was carried out on 100 patients with T2DM at a tertiary care hospital after approval from the institutional ethics committee. Informed consent was obtained from all patients. Patients aged ≥18 years receiving antidiabetic therapy were included in the study. Data, including demographic details, ongoing antidiabetic treatment, and clinical parameters, were recorded and analyzed according to the prescribed drug therapy. Glycemic control was assessed using HbA1c levels, while renal function was evaluated using renal parameters at baseline and during follow-up visits. The data was analyzed using IBM SPSS Statistics for Windows, Version 20 (Released 2011; IBM Corp., Armonk, New York, United States). A student's t-test and ANOVA were applied as appropriate, and a p-value < 0.05 was considered statistically significant.

Results: Patients receiving metformin and gliptin therapy showed a statistically significant reduction (p < 0.05) in HbA1c levels and mean serum urea during the study period. Serum creatinine levels were found to be slightly higher than the normal range in all the groups except metformin-based combination therapy with sulfonylureas and metformin-based combination therapy with sulfonylureas plus voglibose at the first visit. The values dropped down gradually in subsequent visits in all the groups except in the insulin group and the metformin-with-voglibose group, but were not statistically significant. A significant reduction was observed in the group taking metformin with sodium-glucose cotransporter 2 (SGLT2) inhibitors and gliptins. Renal function tests, such as serum urea and serum creatinine levels, showed significant changes during all three subsequent follow-ups.

Conclusion: Proper use of antidiabetic drugs, particularly oral combination therapy, not only improves blood sugar control but also helps in protecting kidney function in diabetic patients. Future investigations with large sample sizes and longer observation periods are warranted to substantiate these findings.

## Introduction

Type 2 diabetes mellitus (T2DM) is a chronic and complex metabolic disorder characterized by insulin resistance and inadequate insulin secretion, leading to elevated blood glucose levels [1]. According to the World Health Organization's diabetes fact sheet, approximately 537 million adults aged 20-79 years are living with diabetes worldwide, representing about one in every 10 adults [2]. The number of adults living with diabetes is projected to increase to 643 million by 2030 and further to 783 million by 2045 [2]. Diabetes accounted for an estimated 6.7 million deaths worldwide in 2021, equating to approximately one death every five seconds [2]. More than 95% of individuals diagnosed with diabetes have T2DM, making it the most prevalent form of the disease worldwide [1]. 

As diabetes mellitus is a chronic and progressive disease, multiple antidiabetic drugs are often required for optimal glycemic control. Metformin is considered the first-line therapy in all patients unless contraindicated or not tolerated. When adequate glycemic control is not achieved with metformin monotherapy, an additional antidiabetic agent is introduced based on the patient’s clinical profile.

Chronic uncontrolled diabetes mellitus can lead to a wide range of debilitating and potentially fatal complications. These include macrovascular complications, such as coronary artery disease, stroke, and peripheral arterial disease, along with microvascular complications, including diabetic retinopathy, nephropathy, and neuropathy [3]. As reported in the International Diabetes Federation (IDF) Diabetes Atlas, nephropathy affects approximately 5.9% of individuals with diabetes in India [4]. The rising incidence of obesity and diabetes has led to diabetic kidney disease becoming one of the foremost causes of chronic kidney disease, significantly increasing the risk of end-stage kidney failure, cardiovascular events, and early death [4]. 

In a clinical trial involving T2DM patients with microalbuminuria, metformin significantly reduced urinary albumin excretion rates [5]. Contrary to the above observations, research involving dipeptidyl peptidase-4 (DPP-4) inhibitors and glucagon-Like peptide-1 (GLP-1) receptor agonists demonstrated no appreciable impact on renal hemodynamic parameters. Additionally, no persistent changes were noted in tubular function or biomarkers indicative of renal damage [6].

Sodium-glucose cotransporter 2 (SGLT2) inhibitors are an emerging class of glucose-lowering agents that target renal tubular glucose reabsorption. Evidence from the EMPA-REG OUTCOME (Empagliflozin Cardiovascular Outcome Event Trial in Type 2 Diabetes Mellitus Patients) trial suggests that empagliflozin offers significant renal protection in individuals with T2DM by delaying the progression of kidney disease and reducing the risk of adverse renal outcomes [7].

Literature search studies have exhibited both renoprotective and non-renoprotective effects of different antidiabetic drugs, and no study comparing the renoprotective effects of antidiabetic drugs was found. Therefore, the present study was designed to compare the effects of these antidiabetic agents on renal function among patients with T2DM.

## Materials and methods

Study design and duration

A hospital-based prospective observational study was carried out in the Department of Pharmacology and the Department of Endocrinology of a tertiary care teaching hospital, Geetanjali Medical College and Hospital, Udaipur, India. The duration of the study was 12 months from April 2024 to March 2025.

Study participants

The study included all adult patients (≥18 years) with T2DM. Patients with T2DM with acute or renal disorders and those with secondary causes of acute kidney injury, such as sepsis, urinary tract infection, or other infections, were excluded.

The sample size was calculated using the formula: $n = \frac{(Z_{1-\alpha/2})^2 \times p \times q}{d^2}$, considering the level of significance of 5% (95% confidence level) and taking the prevalence of diabetic kidney disease of 44% among patients with T2DM in India, based on the results of a meta-analysis done by Hoda F et al., and an absolute precision of 10% [8]. The minimum required sample size was 95 participants. After adjusting for a 5% attrition rate, the final sample size was 100 participants. All patients were followed up at regular intervals of three months for two successive visits over a period of one year.

Ethical consideration

The study was conducted after obtaining approval from the Human Institutional Ethics Committee (GU/HREC/EC/2022/2158) from Geetanjali University. Written informed consent was obtained from all participants prior to their enrollment in the study. Each participant was provided with detailed information regarding the study objectives, procedures, and their rights as research subjects. They were also assured that all personal and study-related information would be kept strictly confidential.

Method of data collection

Detailed information for each participant was recorded in a structured case record form (CRF) (Appendix 1). The CRF included demographic details such as age and sex of the patient. Investigations were systematically documented. Glycemic status was assessed using glycated hemoglobin (HbA1c) levels. The assessment of renal function included blood urea, serum creatinine, and serum uric acid concentrations. Additionally, serum electrolyte levels, including sodium, potassium, and chloride, were recorded for all participants. Antidiabetic drug therapy for each patient was documented, and participants were categorized into groups based on the type of antidiabetic treatment received. All parameters, including glycemic indices, renal function tests, and serum electrolytes, were compared among the different antidiabetic drug groups.

Statistical analysis

The collected data were entered and organized using Microsoft Excel (Microsoft Corporation, Redmond, Washington, United States) for further analysis. The collected data were analyzed using IBM SPSS Statistics for Windows, Version 20 (Released 2011; IBM Corp., Armonk, New York, United States). Categorical variables were represented in the form of percentages and frequencies. Student’s t-test and ANOVA were employed to determine the significance of differences observed between the study groups. A 95% confidence level was used, and a p-value of <0.05 was considered statistically significant.

## Results

**Figure 1:**
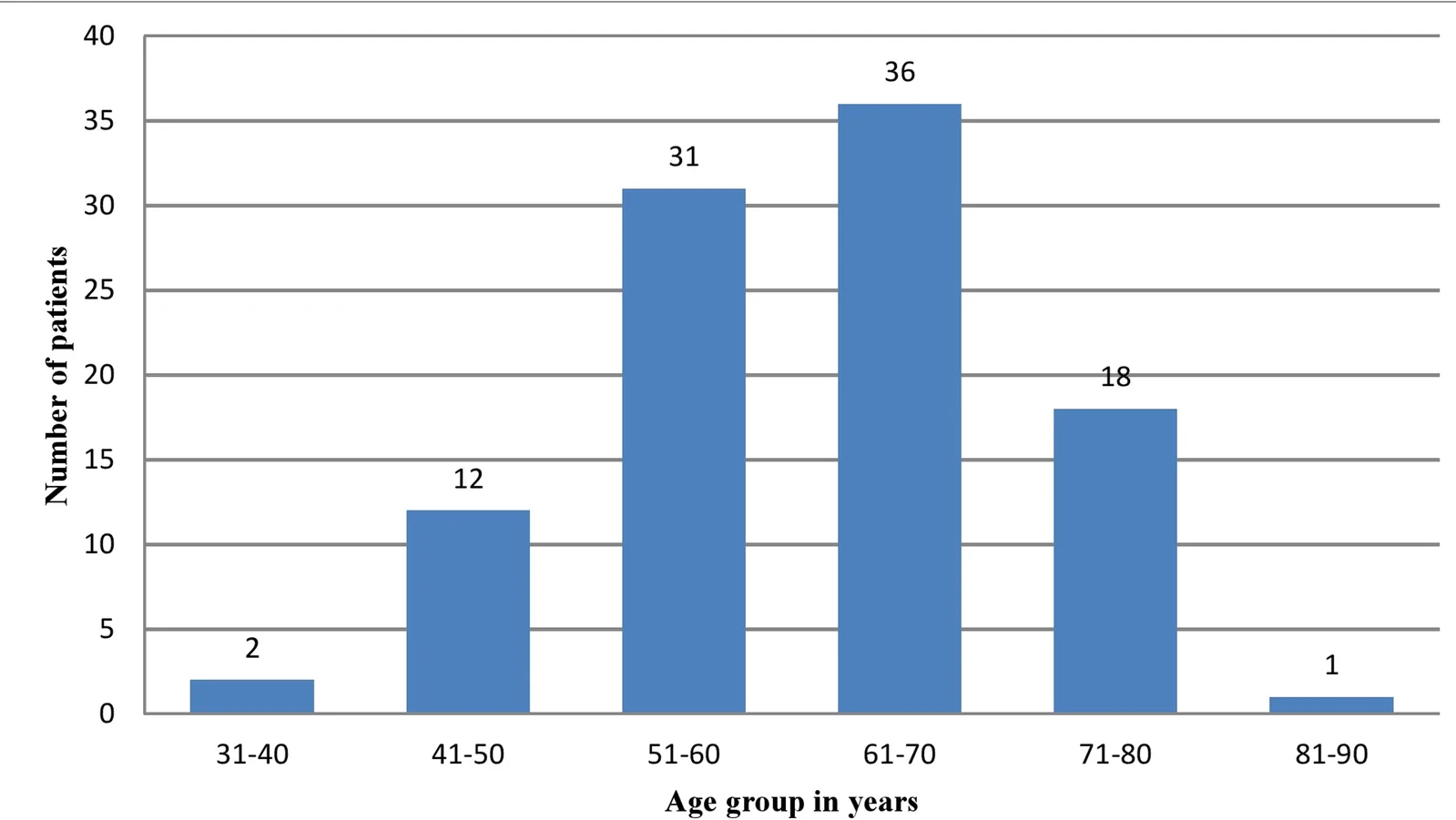
Distribution by age group

**Figure 2:**
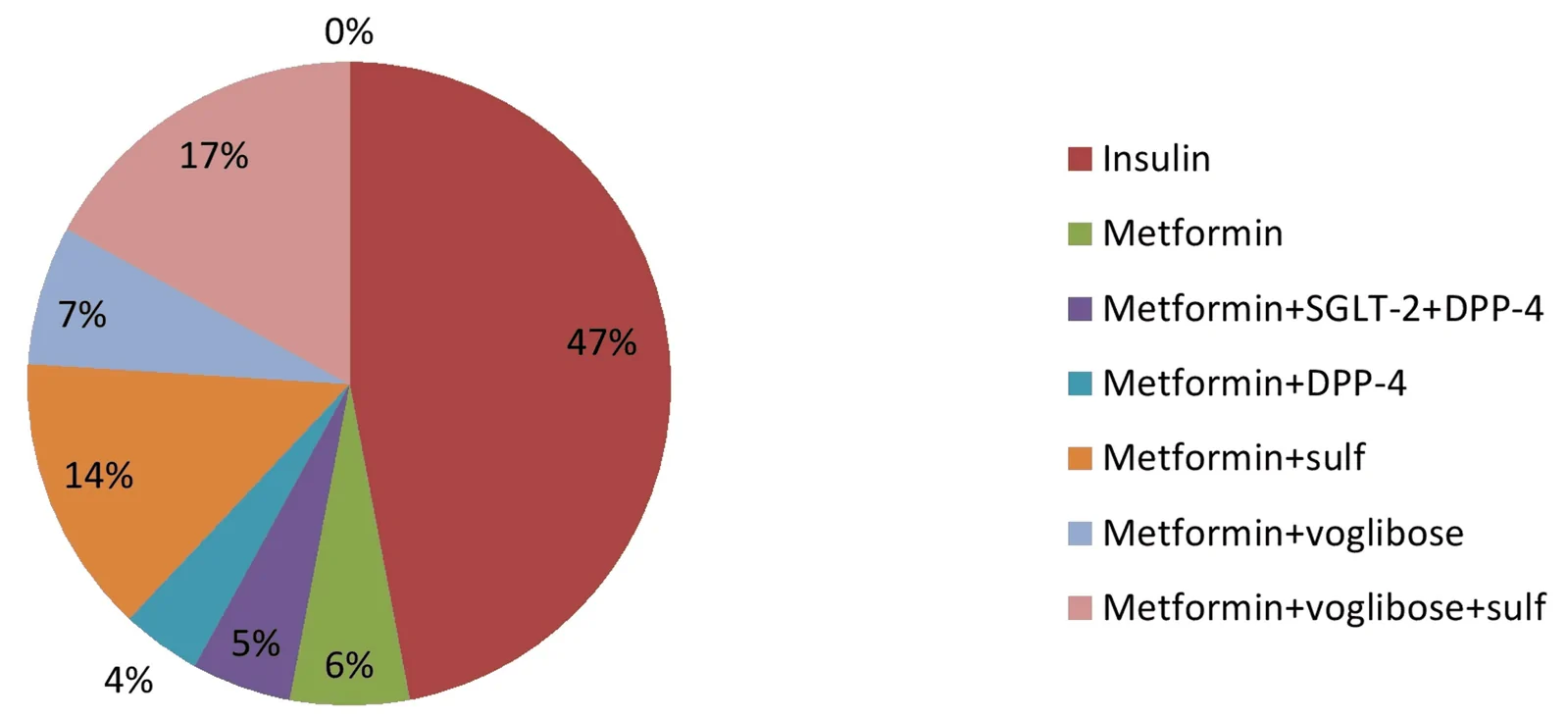
Medication-wise distribution DPP-4: dipeptidyl peptidase-4; SGLT2: sodium-glucose cotransporter 2

Among the 100 patients with T2DM enrolled in the study, 59 (59%) were male, and 41 (41%) were female. The mean age of the study population was 61.71 ± 9.90 years, with 85 (85%) of participants falling within the 51-80 years age group (Figure 1). Around 47 (47%) patients were prescribed insulin therapy either alone or in combination with oral antidiabetic drugs, whereas 53 (53%) patients received only oral antidiabetic drugs. Monotherapy with metformin was prescribed in six (6%) patients, while 94 (94%) patients were treated with two- or three-drug combination regimens. The most commonly prescribed combination therapy was metformin + voglibose + sulfonylurea, administered to 17 (17%) patients, followed by metformin + sulfonylurea, which was prescribed to 14 (14%) patients (Figure 2).

Glycemic status, assessed by HbA1c estimation, showed significant improvement in the group of patients taking metformin with gliptins (p < 0.05). ANOVA revealed a statistically highly significant difference across all three visits (p < 0.001).

Mean serum urea was within the normal range in the visits and across all the groups except in the metformin group, where patients had a mean value that was higher than normal, which reduced to a normal value on the third visit. A significant (p < 0.05) reduction was observed in the patients of the metformin and gliptin groups (Table 1).

**Table 1:** Effect of antidiabetic drugs on serum urea at all visits P-value ≤ 0.05 (statistically significant); one-way analysis of variance (ANOVA) # test is applied

Serum urea				
Medication	1^st^ visit	2^nd^ visit	3^rd^ visit	^#^P-value
	Mean±SD	Mean±SD	Mean±SD	
Insulin (n=47)	46.37±27.59	47.50±31.88	47.23±37.66	>0.05 (NS)
M (n=6)	55.38±53.82	47.58±50.23	41.23±37.31	>0.05 (NS)
m+sulf (n=14)	33.49±17.62	36.37±14.96	40.30±20.34	>0.05 (NS)
m+vog+sulf (n=7)	38.39±18.79	36.36±15.68	35.32±14.96	>0.05 (NS)
m+vog (n=7)	57.17±30.36	50.95±29.27	46.19±19.76	>0.05 (NS)
m+liptin (n=4)	36.38±12.88	33.15±8.11	31.98±10.27	<0.05 (S)
m+dapa+liptin (n=5)	28.30±9.88	27.59±6.03	29.60±8.64	>0.05 (NS)
^#^P-value	<0.001 (HS)	<0.001 (HS)	<0.001 (HS)	

Serum creatinine levels were found to be slightly raised above the normal range in all the groups except two groups: metformin-based combination therapy with sulfonylureas and the group with sulfonylureas and voglibose at the first visit. The values dropped down gradually in subsequent visits in all the groups except in the insulin group and the metformin with voglibose group, but were not statistically significant (p > 0.05). Renal function tests, including serum urea and serum creatinine levels, demonstrated highly significant changes at all three follow-up visits (p < 0.001) (Table 2).

**Table 2:** Effect of antidiabetic drugs on serum creatinine at all visits P-value ≤ 0.05 (statistically significant); one-way analysis of variance (ANOVA) # test is applied

Serum creatinine				
Medication	1^st^ visit	2^nd^ visit	3^rd^ visit	^#^P-value
	Mean±SD	Mean±SD	Mean±SD	
Insulin (n=47)	1.96±2.25	2.02±2.49	2.03±2.27	>0.05 (NS)
M (n=6)	2.10±3.10	1.90±2.57	1.91±2.52	>0.05 (NS)
m+sulf (n=14)	1.06±0.45	1.01±0.33	1.02±0.48	>0.05 (NS)
m+vog+sulf (n=7)	0.88±0.34	0.84±0.18	0.86±0.13	>0.05 (NS)
m+vog (n=7)	1.33±0.53	1.49±0.85	1.42±0.89	>0.05 (NS)
m+liptin (n=4)	1.81±1.03	1.58±1.00	1.42±0.86	>0.05 (NS)
m+dapa+liptin(n=5)	1.52±1.87	1.14±1.11	1.18±1.52	>0.05 (NS)
^#^P value	<0.001 (HS)	<0.001 (HS)	<0.001 (HS)	

Serum uric acid levels were found to be within the normal range in all the groups at all the visits. A significant reduction was observed in the group taking metformin with SGLT2 inhibitors and gliptins (p < 0.05). ANOVA revealed non-significant (p > 0.05) changes at Visit 1, but highly significant changes at Visit 2 and Visit 3 (p < 0.001) (Table 3).

**Table 3:** Effect of antidiabetic drugs on serum uric acid at all visits P-value ≤0.05 (statistically significant); one-way analysis of variance (ANOVA) # test is applied

Serum uric acid				
Medication	1^st^ visit	2^nd^ visit	3^rd^ visit	^#^P-value
	Mean±SD	Mean±SD	Mean±SD	
Insulin (n=47)	6.36±2.18	6.00±2.40	5.64±2.15	>0.05 (NS)
M (n=6)	6.70±2.28	5.49±1.45	5.28±1.65	>0.05 (NS)
m+sulf (n=14)	6.01±1.87	5.80±2.04	6.122.03	>0.05 (NS)
m+vog+sulf (n=7)	6.35±2.14	6.30±1.61	6.38±1.78	>0.05 (NS)
m+vog (n=7)	6.53±2.16	6.14±2.09	5.53±2.51	>0.05 (NS)
m+liptin (n=4)	5.83±1.94	4.54±1.88	4.46±2.28	>0.05 (NS)
m+dapa+liptin(n=5)	5.70±2.38	4.85±1.61	4.40±1.56	<0.05 (S)
^#^P value	>0.05 (NS)	<0.001 (HS)	<0.001 (HS)	

Serum electrolytes, viz., sodium, potassium, and chloride, were analyzed and found to be within the normal range in all the groups at all the visits, and no significant change was observed at the end of the third visit in any group (p > 0.05). ANOVA demonstrated a non-significant difference in serum sodium level at Visit 1 and Visit 2 but showed a significant change at Visit 3 (p > 0.05) (Table 4). Serum chloride levels showed a significant difference at Visit 1 and Visit 3 but were non-significant at Visit 2. Serum potassium levels did not show any significant changes throughout the study period (p > 0.05).

**Table 4:** Comparison of serum sodium levels among antidiabetic drug groups P-value ≤0.05 (statistically significant); one-way analysis of variance (ANOVA) # test is applied

Medication	1^st^ Visit		2^nd^ Visit		3^rd^ Visit		^#^P value
	Mean	SD	Mean	SD	Mean	SD	
Insulin (n=47)	135.38	4.64	135.44	4.74	134.80	4.35	>0.05 (NS)
M (n=6)	134.17	1.94	133.83	3.37	134.17	2.93	>0.05 (NS)
m+sulf (n=14)	134.36	4.22	135.19	3.71	133.94	3.25	>0.05 (NS)
m+vog+sulf (n=7)	134.12	4.12	133.59	5.16	134.18	4.33	>0.05 (NS)
m+vog (n=7)	136.26	6.28	134.94	5.63	141.57	19.03	>0.05 (NS)
m+liptin (n=4)	134.98	5.81	132.80	5.78	132.93	4.07	>0.05 (NS)
m+dapa+liptin (n=5)	133.38	7.47	137.53	5.93	138.18	3.37	>0.05 (NS)
ANOVA ^#^P value	>0.05 (NS)		>0.05 (NS)		<0.05 (S)		

When all the oral antidiabetic drug groups were clubbed together and considered as a single group, all the parameters were compared. Analysis of the insulin group and combined oral antidiabetic drugs group based on S. creatinine values showed that the mean S. creatinine level was higher in the insulin group, which increased slightly in subsequent visits as compared to the oral antidiabetic drugs group, which showed a decrease in subsequent visits, and the difference was statistically significant (p < 0.05) at the second and third visits (Table 5). Analysis of all the parameters, including HbA1c and other renal function tests, among the insulin group and oral antidiabetic drug group was found to be statistically nonsignificant (p > 0.05) (Table 5).

**Table 5:** Comparison between insulin and OHA on the basis of serum electrolytes, RFT, and HbA1c P-value ≤0.05 (statistically significant); unpaired student t-test* is applied RFT: renal function test; OHA: oral hypoglycemic agent

Visit	Medication	S.Urea	S. Creat	S.U.Acid	Na	K+	Cl	HbA1c
1st	Insulin	46.37±27.59	1.96±2.25	6.36±2.18	135.38±4.64	4.85±0.84	101.31±4.57	8.97±2.08
	OHA	40.73±26.22	1.30±1.25	6.30±2.01	134.52±4.55	4.44±0.61	100.82±4.15	9.01±1.96
	*P-value	>0.05 (NS)	>0.05 (NS)	>0.05 (NS)	>0.05 (NS)	<0.05 (S)	>0.05 (NS)	>0.05 (NS)
2nd	Insulin	47.50±31.88	2.02±2.49	6.00±2.40	135.44±4.74	4.52±0.79	101.49±5.20	8.69±1.58
	OHA	38.75±23.08	1.20±1.02	5.86±1.79	134.20±4.62	4.50±0.65	101.36±5.13	8.81±1.99
	*P-value	>0.05 (NS)	<0.05 (S)	>0.05 (NS)	>0.05 (NS)	>0.05 (NS)	>0.05 (NS)	>0.05 (NS)
3rd	Insulin	47.23±37.66	2.03±2.27	5.64±2.15	134.80±4.35	4.65±0.59	100.75±5.27	8.71±1.94
	OHA	38.28±19.72	1.20±1.05	5.79±1.99	135.18±7.79	4.52±0.74	101.45±4.92	8.73±2.11
	*P-value	>0.05 (NS)	<0.05 (S)	>0.05 (NS)	>0.05 (NS)	>0.05 (NS)	>0.05 (NS)	>0.05 (NS)

## Discussion

The present study aims to evaluate the renoprotective effects of antidiabetic drugs over time. With the global rise in diabetes mellitus, there has been a parallel increase in its serious complications, particularly diabetic nephropathy, leading to kidney failure. This growing burden highlights the need to assess commonly used antidiabetic therapies that not only achieve glycemic control but also confer additional renal protection, thereby improving long-term clinical outcomes.

The IDF estimates that 8.8% of adults have diabetes, with slightly higher rates in men than in women [9]. Predominance of the male population was observed in this study. Men have a higher risk of T2DM due to greater insulin resistance, higher fasting glucose, and increased visceral fat, leading to earlier metabolic dysfunction even at lower BMI. In contrast, premenopausal women are relatively protected by estrogen, which improves insulin sensitivity and β-cell function, along with a more favorable subcutaneous fat distribution.

The maximum number of patients in the present study belonged to the elderly population, which is contradictory to the study by Pitale et al. [10], who observed that more than half of the diabetic patients were below 50 years of age. The early onset of T2DM is largely driven by a high prevalence of obesity, which acts as a major risk factor. Additionally, hypertension and dyslipidemia are common comorbid conditions that contribute to metabolic imbalance.

Oral antidiabetic agents are prescribed to patients with preserved endogenous insulin secretion, whereas insulin therapy is initiated when adequate glycemic control is not achieved with oral medications alone. Donnor and Sarkar highlight that insulin plays a direct role in addressing beta-cell dysfunction by boosting natural insulin levels, resulting in greater reductions in blood sugar levels compared to oral medications that enhance insulin sensitivity or secretion [11]. A systematic review further reported that, when used as add-on therapy, basal insulin and oral antidiabetic agents provide similar levels of glycemic control. Insulin therapy demonstrated a statistically significant advantage; the magnitude of this benefit was minimal and considered to have limited clinical relevance [12].

In the present study, insulin was prescribed to a considerable proportion of patients either alone or in combination with oral antidiabetic drugs. This may be attributed to poor glycemic control, progressive β-cell dysfunction, longer duration of diabetes, and associated comorbidities requiring intensive therapy. Although insulin therapy is associated with challenges such as hypoglycemia, weight gain, dose adjustment difficulties, frequent monitoring, and daily injections, it remains highly effective in achieving rapid and adequate glycemic control. The choice of insulin therapy is primarily based on the physician’s clinical judgment and patient-related factors. Patient counseling and acceptance are essential to improve adherence and achieve optimal therapeutic outcomes [10]. In the study conducted by Naeem et al., insulin therapy was associated with a significantly greater reduction in HbA1c levels. However, our study did not demonstrate a similar difference. Instead, a highly significant reduction in HbA1c was observed among patients receiving the combination of metformin and sitagliptin [13].

Kalra et al. reviewed the safety and efficacy of sitagliptin in combination with various OHAs; sitagliptin appears to have a synergistic effect with metformin, probably by preserving β-cell function. The above review also demonstrated that adding sitagliptin to insulin achieves glycemic control, even in patients with inadequate glycemic control with insulin ± metformin [14].

Most of the renal function parameters analyzed were found to be in the normal range, confirming the renoprotection offered by the antidiabetic drugs.

A significant reduction in serum urea level was seen in the group taking metformin with gliptin. Better glycemic control and protective effects due to decreased inflammation, oxidative stress, and fibrosis in the kidney are probably responsible. Alam et al.'s animal study concluded that treatment with sitagliptin prevented inflammation and fibrosis of the kidney by ameliorating elevated oxidative stress in kidney tissues [15]. Long-term high blood glucose levels can induce oxidative stress and proinflammatory cytokine release, which is the cause of diabetic kidney disease. Sitagliptin prevents proapoptotic and inflammatory states affecting renal function improvement in diabetic rat kidneys [16].

Serum creatinine levels showed a slight increase at the third visit compared to the first visit among patients receiving insulin therapy and the combination of metformin with voglibose, but other metformin combination therapies showed a decrease in serum creatinine levels, though statistically not significant. Overall, metformin-based regimens were associated with a stability in serum creatinine levels, which aligns with observations by Zhang et al. [17]. Metformin exhibits renoprotective effects by reducing oxidative stress, inflammation, and fibrosis in the kidneys. It activates AMP-activated protein kinase (AMPK), leading to decreased expression of GRP78, eIF2α, and C/EBP homologous protein (CHOP), thereby mitigating high glucose-induced oxidative stress in human proximal tubular epithelial cells [17]. Additionally, AMPK activation by metformin lowers the production of the receptor for advanced glycation end products (RAGE) and reactive oxygen species (ROS), which in turn suppresses transforming growth factor-β1 (TGF-β1) expression and inhibits renal tubular fibrosis [17]. 

Moreover, uncontrolled diabetes itself damages the kidneys, eventually increasing creatinine, but controlled glucose levels in the study population could be the reason for better renal protection.

Serum creatinine levels were significantly higher in the insulin-treated group than in the oral medication group during both the second and third follow-up visits. Netere et al. reported a rising trend in serum creatinine levels, particularly among patients receiving higher doses of neutral protamine Hagedorn (NPH) insulin. The authors hypothesized that elevated insulin doses may increase the renal clearance burden, as the kidneys play a crucial role in insulin metabolism and excretion, potentially leading to further deterioration of renal function in these patients [18].

Insulin exemplifies the complex and diverse responses of the kidneys, as they play a dual role in both insulin metabolism and insulin action. The kidneys are primarily responsible for the degradation and clearance of circulating insulin, while at the same time remaining highly responsive to insulin's effects throughout various segments of the nephron [19]. The regulation of insulin clearance and its physiological actions by the kidneys is highly complex. Moreover, the wide range of insulin effects is facilitated by the functional specialization of different renal segments, where insulin modulates distinct cellular and metabolic pathways [19].

Skeletal muscle is one of the primary tissues responsive to insulin and plays a central role in postprandial glucose disposal through insulin-mediated glucose uptake. Serum creatinine levels are a product of muscle metabolism, and serum creatinine reflects muscle mass [20].

A generalized decline in serum uric acid levels was observed across most treatment groups; however, a statistically significant reduction was noted among patients receiving the combination of metformin, dapagliflozin, and gliptin. Yip et al. reported that SGLT2 inhibitors are beneficial in reducing serum urate in patients with and without diabetes [21]. According to Chino et al., the extent of enhanced urate excretion by SGLT2 inhibitors harmonizes with the glycosuric effect. SGLT2 inhibition increases tubular glucose, which activates the GLUT9 responsible for the efflux of urate in the proximal tubule [22]. The study revealed a decrease in serum uric acid levels, though not statistically significant, in both the insulin and oral hypoglycemic agent (OHA) groups.

The effect of antidiabetic drugs on serum uric acid levels varies, and different mechanisms of handling uric acid by these drugs have been proposed, which are illustrated by Wang G. et al. and are probably responsible for the overall highly significant reduction in serum uric acid levels observed in the second and third visits [23].

All treatment groups maintained electrolyte levels within the normal range at all the visits. However, at the third follow-up visit, a slight increase in serum sodium levels was observed among patients receiving metformin with voglibose and the combination of dapagliflozin with gliptin. These findings were contradictory to those found by Seven et al. [24].

Several adaptive responses have been suggested to compensate for the increased urinary sodium loss associated with SGLT2 inhibition. Enhanced activation of the renin-angiotensin-aldosterone system may promote greater sodium retention in the distal nephron. By reducing sodium reabsorption in the proximal tubule, SGLT2 inhibitors increase sodium delivery to downstream nephron segments, where compensatory sodium conservation occurs through upregulation of transport processes, including increased activity of the Na⁺-K⁺-2Cl⁻ cotransporter in the loop of Henle and the Na⁺-Cl⁻ cotransporter in the distal convoluted tubule [25].

No significant difference was observed in serum potassium values among the groups during any visit. Serum potassium disorders, either hypokalemia or hyperkalemia, are specifically encountered among the electrolyte disturbances consequent to the progression of disease or may or may not be impacted by antidiabetic drugs. It can also occur due to concomitant medications, as co-morbid conditions are usually present in these patients [26].

Overall serum chloride levels showed significant changes during the first and third visits. Patients receiving metformin-based combination therapy with voglibose plus sulfonylurea and dapagliflozin plus gliptin demonstrated an increase in serum chloride levels during the study period. It was observed by Pawar et al. that the duration of medication usage also influenced electrolyte levels and concluded that short-term use resulted in decreased serum chloride levels and long-term use led to hyperchloremia [27]. The patients included in this study were on the same medications, which exhibited a rise in the values in the present study. A study by Kataoka et al. reported that treatment with SGLT2 inhibitors was associated with an increase in serum chloride levels. The authors proposed several underlying mechanisms, including stimulation of the aldosterone pathway, a decline in serum bicarbonate concentrations due to the buffering effects of organic acid metabolites, and decreased sodium bicarbonate reabsorption. In addition, inhibition of the Na⁺/H⁺ exchanger 3 (NHE3) in the proximal tubules was suggested to promote chloride retention by enhancing chloride reabsorption within the nephron [28].

No statistically significant difference was observed in serum electrolyte levels between patients receiving insulin therapy and those treated solely with oral antidiabetic drugs. Serum potassium concentrations were significantly higher in the insulin-treated group at the initial follow-up visit; however, this difference was transient and was not observed during later follow-up visits. Exogenous insulin increases the activity of the Na+-K+-ATPase pump, and K+ enters hepatic cells and skeletal muscles. Insulin is the most common cause of a decreasing potassium level [29]. Khan et al. suggested that disturbed electrolyte balance is particularly associated with uncontrolled blood sugars in T2DM and even mentioned that serum fasting blood glucose can be used as a predictor for electrolytes [30].

Appropriate use of insulin in optimal doses is essential for effective long-term glycemic management. Furthermore, all oral antidiabetic treatment groups included metformin, which enhances peripheral insulin sensitivity and improves glucose utilization. This may partly explain the relatively maintained serum electrolyte levels observed in the present study.

Overall, the data suggest that the antidiabetic therapies administered in this cohort not only improved glycemic control but also demonstrated renoprotective effects over the three consecutive follow-up periods.

The study had a relatively small sample size (n=100) and a short study duration with a limited follow-up period, which may affect the long-term interpretation of results. Additionally, many patients in the insulin group were also receiving oral antidiabetic drugs, but the effects of these concomitant medications were not analyzed separately in the study.

## Conclusions

This study demonstrated that antidiabetic therapy not only contributes to effective glycemic control but also plays a role in preserving renal function in patients with T2DM. Oral antidiabetic drugs, especially combinations including metformin with gliptins and SGLT2 inhibitors, showed better effects on kidney function parameters like serum urea, creatinine, and uric acid. These drugs also improved glycemic control significantly.

On the other hand, patients receiving insulin showed slightly higher creatinine levels during follow-up, suggesting comparatively less kidney protection than oral drugs. However, most of the kidney function parameters remained within the normal range in all groups, indicating an overall protective effect.

In conclusion, proper use of antidiabetic drugs, particularly oral combination therapy, not only improves blood sugar control but also helps in protecting kidney function in diabetic patients. Further research involving a larger sample population and extended follow-up periods is warranted to validate these findings.

## Figures and Tables

**Figure 3:**
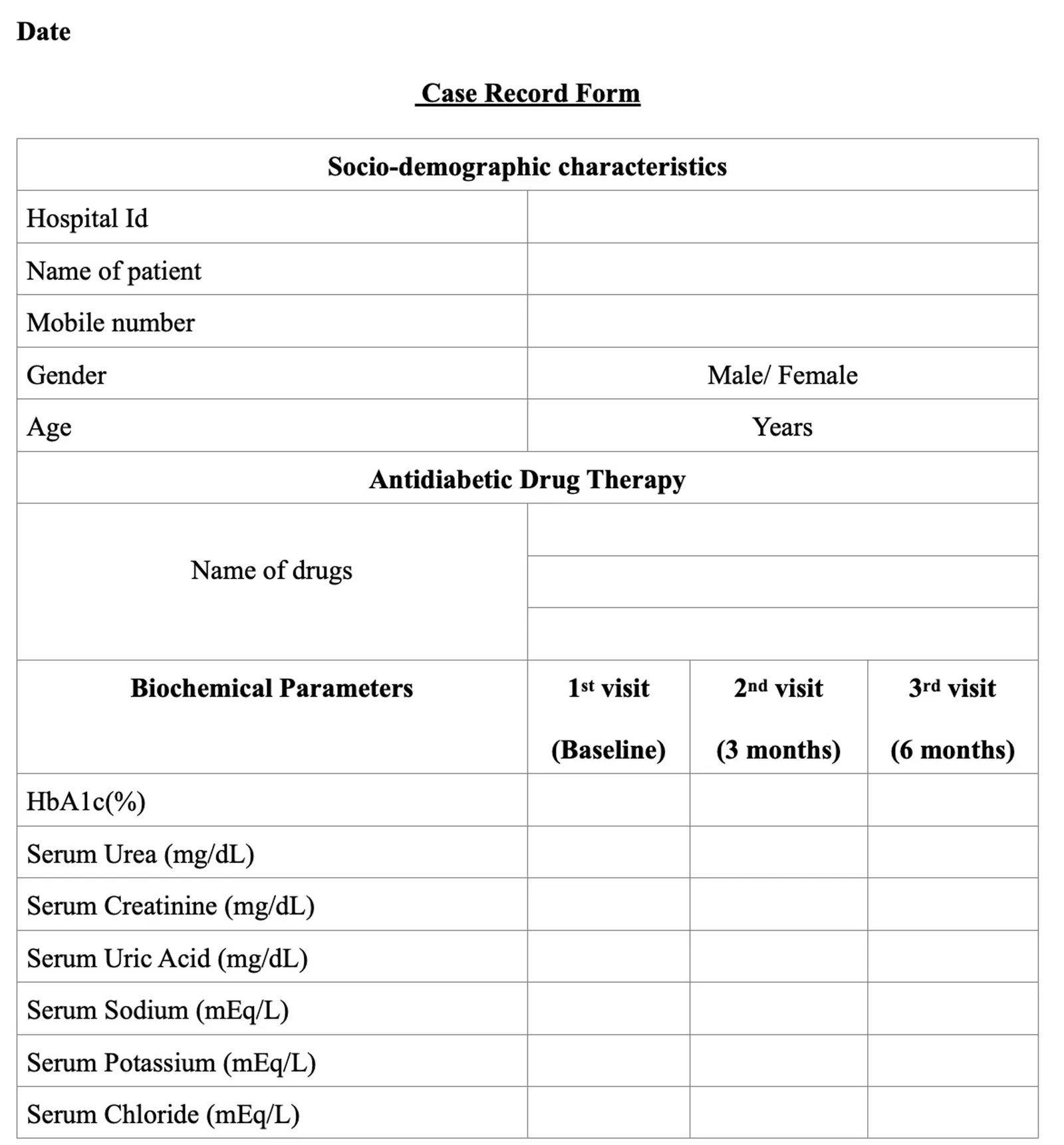
Case record form

## Appendices

Appendix 1
